# Usability and perceived usefulness of patient-centered medication reconciliation using a personalized health record: a multicenter cross-sectional study

**DOI:** 10.1186/s12913-022-07967-7

**Published:** 2022-06-13

**Authors:** Denise J. van der Nat, Victor J. B. Huiskes, Margot Taks, Bart P. H. Pouls, Bart J. F. van den Bemt, Hein A. W. van Onzenoort

**Affiliations:** 1 Department of Clinical Pharmacy, Amphia Hospital, Breda, the Netherlands; 2 Department of Pharmacy, St. Maartenskliniek, Nijmegen, the Netherlands; 3 Department of Pharmacy, Radboud Institute for Health Sciences (RIHS), Radboud University Medical Centre, Nijmegen, the Netherlands; 4 Department of Clinical Pharmacy and Toxicology, Maastricht University Medical Center+, Maastricht, the Netherlands

**Keywords:** Medication reconciliation, Perceived usefulness, Personal health record, Technology acceptance model, Usability

## Abstract

**Background:**

Adoption of a personal health record (PHR) depends on its usability and perceived usefulness. Therefore, we aimed to assess the usability and perceived usefulness of an online PHR used for medication reconciliation and to assess the association between patient-, clinical-, hospital-, and ICT-related factors and the usability and perceived usefulness at both the in- and outpatient clinics.

**Methods:**

A multicenter cross-sectional study was conducted with patients with either an outpatient visit (rheumatology ward) or planned admission in the hospital (cardiology, neurology, internal medicine or pulmonary wards). All patients received an invitation to update their medication list in the PHR 2 weeks prior to their appointment. One month after the hospital visit, PHR-users were asked to rate usability (using the System Usability Scale (SUS)) and perceived usefulness on a 5-point Likert scale. The usability and perceived usefulness were classified according to the adjective rating scale of Bangor et al. The usability was furthermore dichotomized in the categories: low (SUS between 0 and 51) and good (SUS 51–100) usability. Associations between patient-, clinical-, hospital-, and ICT-related factors and the usability and perceived usefulness were analysed.

**Results:**

255 of the 743 invited PHR-users completed the questionnaire. 78% inpatients and 83% outpatients indicated that usability of the PHR was good. There were no significant association between patient-, clinical-, hospital-, and ICT-related factors and the usability of the PHR. The majority of the patients (57% inpatients and 67% outpatients) classified perceived usefulness of the PHR as good, excellent, or best imaginable. Outpatients who also used the PHR for other drug related purposes reported a higher perceived usefulness (adjusted odds ratio 20.0; 95% confidence interval 2.36–170). Besides that, there was no significant association between patient-, clinical-, hospital-, and ICT-related factors and the perceived usefulness of the PHR.

**Conclusions:**

The majority of the patients indicated that the PHR for medication reconciliation was useful and easy to use, but there is still room for improvement. To improve the intervention, further research should explore patients’ barriers and facilitators of using a PHR for medication reconciliation.

**Supplementary Information:**

The online version contains supplementary material available at 10.1186/s12913-022-07967-7.

## Background

For safe and effective clinical care it is essential to know patients actual medication use and to record this in the patient file [1]. It is proven that an accurate medication list can reduce medication errors, especially in high risk patients [2–5]. However, an accurate list of a patients current medication is hardly available, as up to 100% of the in- and outpatients have at least one medication discrepancy, defined as an unexplained difference between the patient’s drug list and the medication a patient is actually taking [6–11].

The gold standard for reducing medication discrepancies is medication reconciliation [12]. The Institute of Healthcare Improvement defines medication reconciliation as “the process of identifying the most accurate list of a patient’s current medicines including the name, dosage, frequency and route — and comparing them to the current list in use, recognizing and documenting any medication discrepancies, finally resulting in a complete list of medications” [13]. A recent study indicated that an online personal health record (PHR), a secure online website that gives patients access to personal health information, is comparable with medication reconciliation for the identification of clinically relevant medication discrepancies in patients’ drug lists [14]. Furthermore, a PHR has the ability to increase patient empowerment which is defined as “the involvement in their own care by individuals, with the goal to make competent, well-informed decisions about their health and health care and take action to support those decisions” [15].

According to the technology acceptance model, acceptance and usage of a PHR depends on the usability and patients’ perceived usefulness of the PHR [16]. Usability of a PHR for the identification of medication discrepancies has previously been investigated with a validated questionnaire (System Usability Scale (SUS) or Post-Study System Usability Questionnaire) in three studies [17–19]. In these studies an acceptable usability of the PHR was observed. Besides that, Marien et al. observed a high perceived usefulness (agreement range: 80–100%) of the PHR [18]. However, in all three studies a limited number of patients derived from a heterogeneous population was included (*N* < 30) and these studies were performed in a single centre [17–19]. As recent studies showed that the capability and motivation of patients using a PHR is influenced by personal factors such as age, educational level, ethnicity and socioeconomic status [15, 20], the usability and perceived usefulness of a PHR used for medication reconciliation may also be associated with patient related factors. Up till now, only one study examined the association between patient-, clinical-, hospital-, and ICT-related factors and the usability and perceived usefulness of such a PHR. This study observed a positive association between the usability and the number of logins to the PHR, but only a limited number of variables was included [18]. Knowledge of these factors may contribute to a more targeted approach for increasing patients’ usage of a PHR. Therefore, the aim of this study was to assess the usability and perceived usefulness of an online PHR used for medication reconciliation and to assess the association between patient-, clinical-, hospital-, and ICT-related factors and the usability and perceived usefulness at both the in- and outpatient clinics.

## Methods

### Setting

A multicenter cross-sectional study was conducted at an outpatient (department of rheumatology of the Sint Maartenskliniek, Nijmegen, the Netherlands) and inpatient clinic (departments of cardiology, neurology, internal medicine and pulmonary of the Amphia Hospital, Breda, the Netherlands). Other than using the same PHR, the in- and outpatient clinics have no relationship to each other.

Patients scheduled for a hospital admission in the period of May to October 2019 or an outpatient rheumatologic visit in the period of October and November 2018 were eligible for this study. Two weeks prior to the planned visit all patients received an invitation to verify their medication list in the online PHR and to adjust their medication list if necessary. About 1 month after the hospital visit, all patients who completed the medication check using the PHR at the Amphia Hospital and 300 random selected PHR-users (of the 2425 PHR-users) of the Sint Maartenskliniek received an invitation by email to participate in this study. The questionnaire was sent using Castor EDC, a cloud-based Electronic Data Capture platform [21]. Two weeks after the first invitation a reminder was sent to complete the questionnaire.

### Ethical considerations

The methods were carried out in accordance with relevant guidelines and regulations like the Declaration of Helsinki and the European General Data Protection Regulation. The study performed at the Amphia Hospital (N2019–0212) was approved by the *Medical Ethics Committee* of Utrecht, the Netherlands, and the study performed at the Sint-Maartenskliniek (2018–4873) was approved by the *Commission on Research Involving Human Subjects* of Arnhem-Nijmegen, the Netherlands. All patients provided written informed consent prior to starting the questionnaire.

### Participants

Eligible patients were included when they were 18 years or older, able to read the Dutch language, had an email address recorded in the electronical heath record and gave informed consent. Patients were excluded if they did not complete the questionnaire in Castor EDC.

### Personal health record

In this study a PHR (Zorgdoc®, Eindhoven, the Netherlands) designed for patients to update their own medication list was evaluated. The PHR system could be accessed with two interfaces: a website for patients and one for healthcare professionals. Both components contain a patient’s medication file; one owned by the patients (Additional file 1) and one by the healthcare professional (Additional file 2). Both components are synchronized, giving the users (patients and professionals) access to the information that has been captured in either file.

The patient’s medication files in the PHR are composed of patient’s input and drug information derived from the Nationwide Medication Record System, a digital nationwide network which exchanges medication dispensing data form all pharmacies in the Netherlands [22]. Patients received an automated invitation to update their medication file approximately 2 weeks prior to their visit. During the verification process, patients were asked to verify the shown medication information derived from the Nationwide Medication Record System. When the patient had finished the verification process, a healthcare professional validated the entered drug information and the drug list was updated in the electronic health record file.

### Outcome measures

The primary outcome of this study was the number of patients that indicated a good usability of the PHR at both the in- and outpatient setting. The secondary outcomes were the score of the perceived usefulness and the association between usability and perceived usefulness and patient-, clinical-, hospital-, and ICT-related factors.

### Measuring usability

**Fig. 1:**
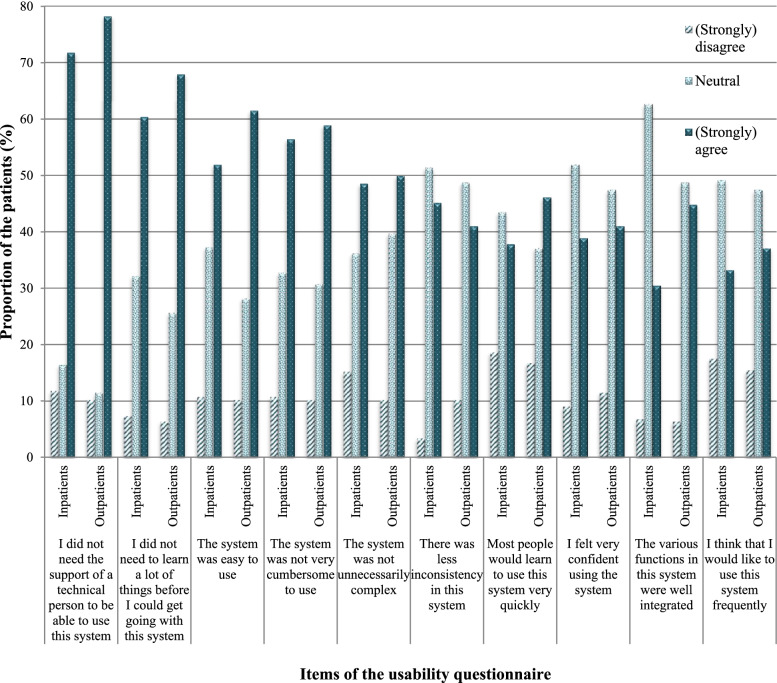
Scores of the items of the usability questionnaire of the personal health record. The data were collected for patients with an outpatient visit (at the rheumatology ward, *N* = 78) or a planned admission in the hospital (at the cardiology, neurology, internal medicine or pulmonary wards, *N* = 177)

Based on the technology acceptance model, the usability and perceived usefulness were examined. Usability of the PHR was measured with the validated SUS translated to Dutch [23, 24]. The SUS questionnaire consists of ten statements (shown in Fig. 1) rated on a 5-point Likert scale. SUS-scores range from 0 (negative) to 100 (positive) and were calculated according to Davis’s guidelines [16]. To calculate the SUS, first the score contributions from each item were summed. Each item’s score contribution ranged from 0 to 4. For the positively worded items of the questionnaire, the score contribution was the scale position minus one point. For the negatively worded items of the questionnaire, the contribution was five points minus the scale position. The sum of all these scores was then multiplied by 2.5 to get the overall SUS-score [16].

The usability was categorized into seven categories using an adjective rating scale as described by Bangor et al.:

- worst imaginable: SUS-score 0–12.5;
- awful: SUS-score 12.6–20.3;
- poor: SUS-score 20.4–35.7;
- ok: SUS-score 35.8–50.9;
- good: SUS-score 51.0–71.4;
- excellent: SUS-score 71.5–85.5; and
- best imaginable: SUS-score 85.6–100 [25].

As we expected no linear relation between the SUS-score and the patient-, clinical-, hospital-, and ICT-related factors, we dichotomized these seven categories in the categories: low (SUS between 0 and 51) and good (SUS 51–100) usability [26].

### Measuring perceived usefulness

**Fig. 2:**
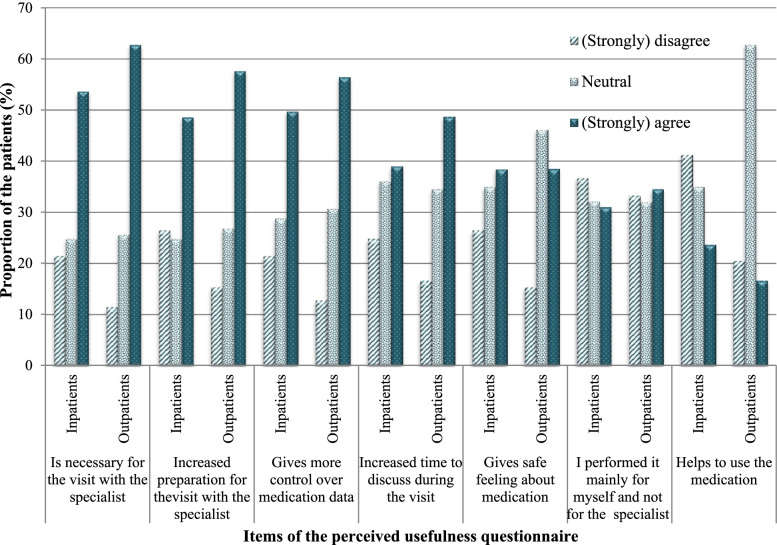
Scores of the items of the perceived usefulness questionnaire of the personal health record. The data were collected for patients with an outpatient visit (at the rheumatology ward, *N* = 78) or a planned admission in the hospital (at the cardiology, neurology, internal medicine or pulmonary wards, *N* = 177)

Perceived usefulness was assessed with the perceived usefulness questionnaire by Davis et al. as basis and adjusted to fit the purpose of our PHR [16]. The created questionnaire consisted of two parts rated on a 5-point Likert scale. The first part contained seven questions (shown in Fig. 2) focusing on the perceived usefulness of the PHR itself and part two consisted of three questions which explored the perceived usefulness of the PHR compared to the gold standard, medication reconciliation performed by a healthcare professional. In our research, medication reconciliation was performed by a pharmacy technician who compiled the best possible medication history according to the standard operating procedure according to the World Health Organization [12].

Like the SUS, a score of the perceived usefulness was calculated by summing the score contributions (scale position minus one point) of the first seven questions of the perceived usefulness questionnaire. Based on the method described by ‘measuring usability’, the sum of the seven scores was multiplied by 3.57 (to reach a maximum score of 100) to get the overall perceived usefulness score. As there is no validated categorization of the perceived usefulness, the adjective rating scale of Bangor et al. was applied [25].

### Data collection

The data were collected in Castor EDC. Based on literature [15, 18, 27], patient-, clinical-, hospital-, and ICT-related factors were collected (Table 1). Over-the-counter medication was defined as drugs that were sold to patients without a doctor’s prescription [28]. Known comorbidities were extracted from the EHR which were classified according to the International Classification of Diseases-10 [29]. All diagnoses in the patient’s medical record with the status ‘current’ were taken into account. To make sure that the list was complete and correct, the information was checked and supplemented with comorbidities based on drug information of the best possible medication history.

**Table 1:** Collected patient-, clinical-, hospital-, and ICT-related factors. During the study, the following patient-, clinical-, hospital-, and ICT-related factors were collected from the patient, electronical health record or personal health record

Variable	Source of information	Additional explanation
**Patient-related factors**		
**Patient’s age**	Patient	–
**Patient’s gender**	Patient	–
**Patient’s highest education level based on the Dutch standard educational classification**	Patient	The education level was based on the Dutch standard educational classification.
**Patient’s experience with digital devices (including device usage time)**	Patient	Experience with digital devices was scored from low (score 1) to high (score 10). The hours a week of private internet use was classified into: 0–7, 7–14, 14–28 h or more than 28 h.
**Patient’s knowledge of the indication of each drug**	Patient	Patients indicated if they knew the indication(s) of all their drugs, a part of their drugs of none of their drugs.
**Clinical-related factors**		
**The number of drugs on the BPMH**	Personal health record	The number of drugs was determined from the BPMH created prior to the hospital visit.
**The number of specialism-related drugs on the BPMH**	Personal health record	Three pharmacists composed a list of ATC-codes which was related with the admitted medical specialism (cardiology, neurology, internal medicine, rheumatology and pulmonary ward). Subsequently, for each patient the number of drugs categorized to the ATC-codes related with their admitted department was counted.
**The number of over-the-counter medications**	Patient	Drugs which were sold to patients without a doctor’s prescription were classified as over-the-counter medication.
**The number of changes in patient’s drug list in the last 12 months**	Personal health record	The number of changes was calculated from the registered period of use of the drugs in the last 12 months prior to the hospital visit. Changes in the patient’s drug list included changes in dose and frequency.
**The number of known comorbidities**	Personal health record	All diagnoses in the patient’s medical record with the status ‘current’ were taken into account. To make sure that the list was complete and correct, the information was checked and supplemented with comorbidities based on drug information of the BPMH.
**The number of years under treatment of the specialist**	Electronic health record	The number of years between the first registered contact with the specialist and the current hospital visit was calculated.
**The number of different prescribers (except for the general practitioner)**	Patient	–
**Hospital-related factors**		
**The type of prescriber during the outpatient rheumatologic visit**	Electronic health record	The outpatient visit was performed by a physician assistant or rheumatologist.
**The reason for the outpatient rheumatologic visit**	Patient	The reasons for the visit were categorized into: diagnosis, new disease, follow-up appointment or other reasons.
**The number of outpatient visits to the specialist in the last 12 months**	Electronic health record	Registered outpatient visits to the admitted medical department were counted.
**The number of hospitalization at the admitted department in the last 12 months**	Electronic health record	Registered hospitalizations to the admitted medical department in the Amphia Hospital were counted.
**ICT-related factors**		
**The number of logins to the PHR 12 months before the appointment**	Personal health record	–
**The type of device to log in to the PHR**	Patient	The type of device was categorised into: computer, tablet or smartphone.
**The availability of data import from the NMRS in the PHR**	Personal health record	The NMRS contain patients’ medication dispensing data form all pharmacies in the Netherlands. If data from the NMRS is not available, inpatients see a blank medication list. If the patient had used the PHR before, the previous medication list is shown. If data from the NMRS is not available at the outpatient clinic, patients see their drug list registered in the EHR.
**The available time for patients to log in to the PHR**	Personal health record	The available time for patients to log in to the PHR was the number of days between sending the invitation of the PHR and the day prior to the hospital visit.
**The number of days between sending the invitation and the patients login to the PHR**	Personal health record	–
**The usage of the PHR with or without help from others**	Patient	–
**The use of other drug-related functions of the PHR**	Patient	Other drug-related functions were classified as requesting a prescription refill and printing a current medication list.
**The proportion of logins to the PHR up to 12 months after the hospital visit**	Personal health record	The proportion of logins was calculated as the number of logins divided by the number of sent invitations.

*Abbreviations*: *ATC-classification* anatomical therapeutic chemical classification, *BPMH* best possible medication history, *EHR* electronic health record, *MR* medication reconciliation, *PHR* personal health record, *NMRS* nationwide medication record system

### Statistical analysis

First, descriptive analyses were performed to describe usability and perceived usefulness of the PHR at both the in- and outpatient setting. Descriptive statistics were provided using mean (± standard deviation [SD]) or median (interquartile range [IQR]) values depending on the (non-)parametric distribution of measured variables.

In order to examine the association of the patient-, clinical-, hospital-, and ICT-related factors and the usability (low usability versus good usability) and perceived usefulness (first quartile (Q1) versus fourth quartile (Q4) of the perceived usefulness score), the factors were first entered into a univariate logistic regression model after which the significant risk factors (*P* < .1) were selected and incorporated into the full model logistic regression analyses. An independent-samples nonparametric test was used to compare patient characteristics between the in- and outpatient setting. To determine the reliability of our results, an independent-sample nonparametric test was used to compare the age, number of drugs and number of logins to the PHR between the participating and non-participating patients. Furthermore, a Chi-square test was used to compare the gender and the admitted medical department between the participating and non-participating patients. Results were considered statistically significant at *P* < .05. Data were analysed using IBM SPSS Statistics software version 25.

## Results

About half of the patient (43% inpatients and 46% outpatients) accepted the invitation to perform medication reconciliation by using a PHR (Additional file 3). All patients who started using the PHR also finished the medication reconciliation process. Of the PHR-users, 743 patients were invited for the questionnaire of which 255 (34%) patients completed the questionnaire. The survey response rate was slightly higher at the inpatient setting (40%) compared to the outpatient setting (26%). Responders had a median age of 65 and 59 (for inpatients and outpatients, respectively), covered all educational levels, were fairly experienced with digital devices, used a median number of 7 and 5 drugs (for inpatients and outpatients respectively), were very knowledgeable regarding their medications and had many changes to their drugs in the last 12 months (Table 2). Non-participating outpatients were significantly younger (median age 48; IQR: 36–60, *P* < .001) and used less drugs (median 2; IQR: 1–6, *P* < .001) than participating outpatients. Furthermore, non-participating inpatients were more often a female (43% versus 31%, *P* = .02) compared to the participating inpatients.

**Table 2:** Characteristics of patients with an outpatient visit (at the rheumatology ward) or a planned admission in the hospital (at the cardiology, neurology, internal medicine or pulmonary wards). More detailed information about the (collection of the) characteristics are described in Table 1

	Inpatients (***N*** = 177)	Outpatients (***N*** = 78)
**Age (years, median (IQR))**	65 (57–71)	59 (50–65)
**Male, n(%)**	122 (69)	20 (26)
**Highest educational level, n(%)**		
Primary school	7 (4)	3 (4)
Secondary school: low level	26 (15)	10 (13)
Secondary vocational education	61 (35)	35 (45)
Secondary school: high level	20 (11)	9 (12)
Universities of applied sciences	48 (27)	19 (24)
University	15 (9)	2 (3)
**Hours a week of private internet use, n(%)**		
0–7	73 (41)	42 (54)
7–14	59 (33)	25 (32)
14–28	37 (21)	10 (13)
> 28	8 (5)	1 (1)
**Experience with digital devices (score from low (1) to high (10)), n(%)**		
1–2	12 (7)	3 (4)
3–4	10 (6)	6 (8)
5–6	39 (22)	16 (21)
7–8	81 (46)	42 (54)
9–10	35 (20)	11 (14)
**Patients with knowledge about the indication(s) of their drug(s), n(%)**	161 (91)	73 (94)
**Number of drugs on the BPMH, median (IQR)**	7 (3–10)	5 (3–7)
**Number of specialism-related drugs on the BPMH, median (IQR)**	4 (2–5)	1 (0–2)
**Number of OTC medication, median (IQR)**	0 (0–1)	1 (0–2)
**Number of changes in patient’s drug list in the last 12 months, median (IQR)**	16 (7–28)	13 (7–22)
**Number of known comorbidities, median (IQR)**	4 (2–6)	3 (2–5)
**Number of years under treatment of the specialist, median (IQR)**	4 (0–10)	0 (0–0)
**Number of different prescribers (except for the general practitioner), median (IQR)**	1 (1–2)	2 (1–2)
**Type of prescriber, n(%)**		
Physician assistant	–	12 (15)
Rheumatologist	–	66 (85)
**Reason for the outpatient rheumatologic visit, n(%)**		
Diagnosis	–	48 (62)
New disease	–	10 (13)
Follow-up appointment	–	7 (9)
Other	–	13 (17)
**Number of outpatient visits to the specialist in the last 12 months, median (IQR)**	3 (2–6)	0 (0–0)
**Number of hospital admissions at the admitted department in the last 12 months, median (IQR)**	0 (0–1)	–
**Device used to log in to the PHR, n(%)**		
Computer	115 (65)	46 (59)
Tablet	26 (15)	14 (18)
Smartphone	36 (20)	18 (23)
**Data import of the NMRS in the PHR, n(%)**	156 (88)	64 (82)
**Available time for patients to connect with the PHR (days, median (IQR))**	7 (5–8)	13 (11–13)
**Number of days between sending the invitation and the patients login to the PHR, median (IQR))**	0 (0–1)	3 (2–7)
**PHR used without help from others, n(%)**	173 (98)	78 (100)
**PHR used for other purposes, n(%)**		
Printing a current medication list	–	14 (18)
Requesting a prescription refill	–	4 (5)
**Number of logins to the PHR 12 months before the visit, n(%)**		
0	167 (94)	74 (95)
1	10 (6)	4 (5)
**Percentage of logins to the PHR up to 12 months after the appointment, n(%)**		
No invitation of the PHR received	153 (86)	38 (49)
0%	10 (6)	13 (17)
1–24%	0 (0)	0 (0)
25–50%	2 (1)	1 (1)
51–75%	3 (2)	8 (10)
76–100%	9 (5)	18 (23)

*Abbreviations*: *BPMH* best possible medication history, *IQR* interquartile range, *MR* medication reconciliation, *NMRS* nationwide medication record system, *OTC medication* over-the-counter medication, *PHR* personal health record

### Usability

The majority of the patients (78% inpatients and 83% outpatients) indicated that the usability of the PHR for medication reconciliation was good or even better (Table 3).

**Table 3:** Usability of a personal health record classified according to the adjective rating scale of the System Usability Scale. The data were collected for patients with an outpatient visit (at the rheumatology ward) or a planned admission in the hospital (at the cardiology, neurology, internal medicine or pulmonary wards)

Adjective rate of the usability of the personal health record	Inpatients (***N*** = 177) n(%)	Outpatients (***N*** = 78) n(%)
**Worst imaginable**	1 (1)	0 (0)
**Awful**	1 (1)	0 (0)
**Poor**	6 (3)	3 (4)
**Ok**	32 (18)	11 (14)
**Good**	93 (53)	38 (49)
**Excellent**	37 (21)	23 (30)
**Best imaginable**	7 (4)	3 (4)

The mean SUS-score of all in- and outpatients was 63 (SD: 14) and 64 (SD: 14) respectively. In- and outpatients most often (strongly) agreed with the item ‘I did not need the support of a technical person to be able to use the PHR’ (72 and 78% respectively) followed by ‘I did not need to learn a lot of things before I could get going with this system’ (61 and 68% respectively). Patients most often (strongly) disagreed with the item ‘most patients would learn to use this system very quickly’ (19 and 17% respectively) followed by ‘I think that I would like to use this system frequently’ (18 and 15% respectively) (Fig. 1). Patients noted in their remarks that the PHR was especially difficult to use for patients with low literacy and the elderly.

### Association between patient-, clinical-, hospital-, and ICT-related factors and the usability of a personal health record used for medication reconciliation

#### Inpatient clinic

In the univariate analyse, four factors were significantly (*P* < .05) associated with a good usability. These concerned experience with digital devices, patients’ knowledge about the indication(s) of their drug(s), number of different prescribers and the device used to log in to the PHR (Table 4). In the adjusted analyses, there was no significant association between usability and patient-, clinical-, hospital-, and ICT-related factors (Table 4).

**Table 4:** Patient-, clinical-, hospital-, and ICT-related factors associated with a good usability of a PHR. The data were collected for patients with an outpatient visit (at the rheumatology ward, *N* = 78) or a planned admission in the hospital (at the cardiology, neurology, internal medicine or pulmonary wards, *N* = 177)

	Inpatients (***N*** = 177)		Outpatients (***N*** = 78)	
	Crude OR (95%CI)	Adjusted OR^**a (**^95%CI)	Crude OR (95%CI)	Adjusted OR^**b**^ (95%CI)
**Age**	0.99 (0.96–1.02)	–	0.96 (0.91–1.01)	–
**Gender**				
Male	Referent	–	Referent	–
Female	0.79 (0.38–1.67)	–	2.68 (0.80–9.01)	–
**Highest educational level**				
Primary school	Referent	–	Referent	–
Secondary school: low level	0.32 (0.03–3.04)	–	1.17 (0.07–18.3)	–
Secondary vocational education	0.43 (0.05–3.85)	–	3.00 (0.23–39.6)	–
Secondary school: high level	1.50 (0.12–19.6)	–	0.63 (0.04–9.65)	–
Universities of applied sciences	0.72 (0.08–6.77)	–	9.00 (0.39–207)	–
University	1.08 (0.08–14.4)	–	–	–
**Hours a week of private internet use**				
0–7	Referent	–	Referent	Referent
7–14	1.28 (0.56–2.93)	–	4.60 (0.94–22.6)**	4.03 (0.80–20.2)**
14–28	1.02 (0.41–2.56)	–	–	–
> 28	2.29 (0.26–19.9)	–	–	–
**Experience with digital devices**	1.39 (1.18–1.64)*	–	1.36 (1.01–1.83)*	1.26 (0.93–1.72)
**Patients with knowledge about the indication(s) of their drug(s)**				
Yes	Referent	–	Referent	–
No	–	–	0.36 (0.03–4.26)	–
Partly	0.30 (0.10–0.87)*	–	–	﻿–
**Number of drugs on the BPMH**	0.94 (0.87–1.02)	–	1.07 (0.91–1.25)	–
**Number of specialism-related drugs on the BPMH**	1.00 (0.87–1.15)	–	1.60 (0.89–2.90)	–
**Number of over-the-counter medication**	0.95 (0.78–1.16)	–	0.85 (0.61–1.19)	–
**Number of changes in patient’s drug list in the last 12 months**	1.00 (0.97–1.01)	–	1.00 (0.96–1.04)	–
**Number of known comorbidities**	0.90 (0.79–1.02)	–	0.96 (0.74–1.26)	–
**Number of years under treatment of the specialist**	0.96 (0.90–1.03)	–	0.74 (0.47–1.17)	–
**Number of different prescribers**	0.69 (0.49–0.98)*	–	1.09 (0.65–1.84)	–
**Type of prescriber**				
Physician assistant	–	–	–	–
Rheumatologist	–	–	–	–
**Reason for the outpatient visit**				
Diagnosis	–	–	Referent	–
Follow-up appointment	–	–	0.58 (0.10–3.47)	–
New disease	–	–	–	–
Other	–	–	0.77 (0.18–3.38)	–
**Number of outpatient visits to the specialist in the last 12 months**	1.03 (0.94–1.14)	–	0.68 (0.34–1.36)	–
**Number of hospital admissions at the admitted department in the last 12 months**	1.10 (0.71–1.69)	–	–	–
**Device used to log in to the PHR**				
Computer	Referent	–	Referent	﻿–
Tablet	0.37 (0.15–0.96)*	–	1.67 (0.32–8.70)	﻿–
Smartphone	0.40 (0.17–0.93)*	–	2.22 (0.44–11.3)	﻿–
**Data input from the NMRS in the PHR**				
No	Referent	–	Referent	–
Yes	0.78 (0.25–2.48)	﻿–	0.72 (0.14–3.66)	–
**Available time for patients to log in to the PHR**	0.92 (0.82–1.04)	–	1.08 (0.75–1.56)	–
**Number of days between sending the invitation and the patient login to the PHR**	1.03 (0.80–1.33)	–	0.94 (0.77–1.16)	–
**Help received for using the PHR**				
Yes	Referent	–	Referent	–
No	3.55 (0.48–26.1)	–	–	–
**PHR used for other drug-related purposes**				
No	–	﻿–	Referent	–
Yes	–	–	4.33 (0.53–35.8)	–
**Percentage of logins 12 months before the hospital visit**	1.01 (0.98–1.04)	–	1.00 (0.96–1.05)	–
**Percentage of logins 12 months after the hospital visit**	1.02 (1.00–1.05)**	1.01 (0.95–1.07)	1.00 (0.98–1.02)	–

*Abbreviations*: *BPMH* best possible medication history, *NMRS* nationwide medication record system

* *P* < .05

** *P* < .1

^a^The significant risk factors (*P* < .1): experience with digital devices, patient’s knowledge about the indication(s) of their drug(s), the number of different prescribers, device used to log in to the PHR, and the percentage of logins 12 months after the hospital visit were selected and incorporated in the full model logistic regression analyses

^b^The significant risk factors (*P* < .1): hours a week of private internet use and experience with digital devices were selected and incorporated in the full model logistic regression analyses

#### Outpatient clinic

In the univariate analyse, experience with digital devices and hours a week of private internet use were significantly (*P* < .05) associated with a good usability (Table 4). In the adjusted analyses, there was no significant association between usability and patient-, clinical-, hospital-, and ICT-related factors (Table 4).

### Perceived usefulness

The mean score of the perceived usefulness of all in- and outpatients was 53 (SD: 18) and 58 (SD: 18) respectively. The majority of the patients (57% inpatients and 67% outpatients) indicated that the perceived usefulness of the PHR for medication reconciliation was good or even better (Table 5). Most in- and outpatients (76 and 78%, respectively) agreed that the PHR yielded at least one benefit (out of seven) with regard to their visit to the physician (Fig. 2). In- and outpatients most often (strongly) agreed with the item ‘necessary for the visit with the specialist’ (54 and 63%, respectively) followed by ‘increased preparation for the visit’ (49 and 58%, respectively) and ‘gives more control over medication data’ (50 and 56%, respectively).

**Table 5:** Perceived usefulness of a personal health record classified according to the adjective rating scale of the System Usability Scale. The data were collected for patients with an outpatient visit (at the rheumatology ward) or a planned admission in the hospital (at the cardiology, neurology, internal medicine or pulmonary wards)

Adjective rate of the perceived usefulness of the personal health record	Inpatients (***N*** = 177) n(%)	Outpatients (***N*** = 78) n(%)
**Worst imaginable**	4 (2)	3 (4)
**Awful**	4 (2)	0 (0)
**Poor**	32 (18)	5 (6)
**Ok**	36 (20)	18 (23)
**Good**	80 (45)	42 (54)
**Excellent**	14 (8)	6 (8)
**Best imaginable**	7 (4)	4 (5)

### Association between patient-, clinical-, hospital-, and ICT-related factors and the perceived usefulness of a personal health record used for medication reconciliation

#### Inpatient clinic

In the univariate analyse, the number of outpatient visits to the specialist in the last 12 months and the number of hospital admissions at the admitted department in the last 12 months were significantly (*P* < .05) associated with perceived usefulness (SUS ≤ 39.3 (Q1) versus SUS ≥ 67.8 (Q4)). In the adjusted analyses, there was no significant association between perceived usefulness and patient-, clinical-, hospital-, and ICT-related factors (Table 6).

**Table 6:** Patient-, clinical-, hospital-, and ICT-related factors associated with the perceived usefulness of a personal health record. The data were collected for patients with an outpatient visit (at the rheumatology ward, *N* = 78) or a planned admission in the hospital (at the cardiology, neurology, internal medicine or pulmonary wards, *N* = 177)

	Inpatients (***N*** = 177)		Outpatients (***N*** = 78)	
	Crude OR (95%CI)	Adjusted OR^﻿a^ (95%CI)	Crude OR (95%CI)	Adjusted OR^**b**^ (95%CI)
**Age**	1.02 (0.98–1.06)	–	0.99 (0.95–1.04)	–
**Gender**				
Male	Referent	–	Referent	–
Female	1.18 (0.49–2.82)	–	1.05 (0.31–3.57)	–
**Highest educational level**				
Primary school	Referent	–	–	–
Secondary school: low level	2.60 (0.14–50.0)	–	–	–
Secondary vocational education	1.00 (0.06–17.5)	–	–	–
Secondary school: high level	0.80 (0.04–17.2)	–	–	–
Universities of applied sciences	0.63 (0.04–11.2)	–	–	–
University	0.50 (0.02–11.1)	–	–	–
**Hours a week of private internet use**				
0–7	Referent	–	Referent	–
7–14	0.43 (0.16–1.10)**	0.46 (0.17–1.24)	1.07 (0.32–3.63)	–
14–28	0.61 (0.18–2.02)	0.57 (0.16–1.96)	2.68 (0.45–16.1)	–
> 28	0.70 (0.13–3.90)	0.73 (0.12–4.64)	–	–
**Experience with digital devices**	1.00 (0.82–1.21)	﻿–	0.96 (0.73–1.27)	–
**Patients with knowledge about the indication(s) of their drug(s)**				
Yes	Referent	–	Referent	–
No	–	–	0.48 (0.04–5.65)	–
Partly	0.50 (0.09–2.87)	–	–	–
**Number of drugs on the BPMH**	1.03 (0.95–1.12)	–	1.10 (0.96–1.25)	–
**Number of specialism-related drugs on the BPMH**	1.01 (0.87–1.18)	–	1.35 (0.87–2.11)	–
**Number of over-the-counter medication**	0.97 (0.72–1.29)	–	1.11 (0.71–1.74)	–
**Number of changes in patient’s drug list in the last 12 months**	1.01 (0.99–1.04)	–	0.99 (0.96–1.03)	–
**Number of known comorbidities**	1.08 (0.93–1.25)	–	1.16 (0.90–1.51)	–
**Number of years under treatment of the specialist**	–	–	–	–
**Number of different prescribers**	0.81 (0.54–1.21)	–	1.50 (0.89–2.54)	–
**Type of prescriber**				
Physician assistant	–	–	Referent	–
Rheumatologist	–	–	0.64 (0.16–2.58)	–
**Reason for the outpatient visit**				
Diagnosis	–	–	Referent	–
Follow-up appointment	–	–	0.63 (0.09–4.28)	–
New disease	–	–	2.81 (0.49–16.2)	–
Other	–	–	0.47 (0.10–2.22)	–
**Number of outpatient visits to the specialist in the last 12 months**	0.87 (0.76–0.98)*	0.90 (0.78–1.03)	1.07 (0.46–2.48)	–
**Number of hospital admissions at the admitted department in the last 12 months**	0.43 (0.22–0.84)*	0.51 (0.24–1.09)**	–	–
**Device used to log in to the PHR**				
Computer	Referent	–	Referent	–
Tablet	0.47 (0.14–1.57)	–	1.43 (0.32–6.39)	–
Smartphone	0.60 (0.20–1.77)	–	1.53 (0.42–5.47)	–
**Data input from the NMRS in the PHR**				
No	Referent	–	Referent	–
Yes	0.82 (0.29–2.36)	–	0.75 (0.15–3.73)	–
**Number of days between sending the invitation and the patient login to the PHR**	1.01 (0.73–1.38)	–	1.01 (0.83–1.22)	–
**PHR used for other drug-related purposes**				
No	–	–	Referent	Referent
Yes	–	–	20.00 (2.36–170)*	20.0 (2.36–170)*
**Percentage of logins 12 months before the hospital visit**	1.02 (0.99–1.06)	–	1.03 (0.98–1.07)	–
**Percentage of logins 12 months after the hospital visit**	0.98 (0.94–1.01)	–	1.00 (0.99–1.02)	–

*Abbreviations*: *BPMH* best possible medication history, *NMRS* nationwide medication record system

^a^The significant risk factors (*P* < .1): hours a week of private internet use, the number of outpatient visits to the specialist in the last 12 months, and the number of hospital admissions at the admitted department in the last 12 months were selected and incorporated in the full model logistic regression analyses

^b^The significant risk factor (*P* < .1) ‘PHR used for other drug-related purposes’ was selected and incorporated in the full model logistic regression analyses

* *P* < .05

** *P* < .1

#### Outpatient clinic

When outpatients with a perceived usefulness score of ≤ 50.0 (Q1) were compared with patients with a score of ≥ 67.8 (Q4), we observed that outpatients who also used the PHR for other drug related purposes, reported more often a higher perceived usefulness (adjusted odds ratio 20.0; 95% confidence interval 2.36–170) (Table 6).

### Perceived usefulness of the personal health record compared to the gold standard, medication reconciliation by a healthcare professional

**Fig. 3:**
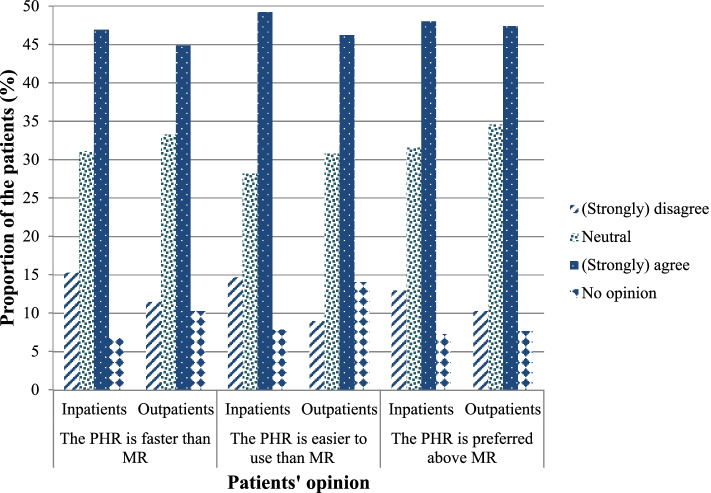
Utility of an online personal health record (PHR) compared to the gold standard. The data were collected for patients with an outpatient visit (at the rheumatology ward, *N* = 78) or a planned admission in the hospital (at the cardiology, neurology, internal medicine or pulmonary wards, *N* = 177). The gold standard was medication reconciliation (MR) performed by a healthcare professional

When the PHR was compared to the gold standard, medication reconciliation performed by a healthcare professional, 48% of the inpatients and 47% of the outpatients preferred the PHR above medication reconciliation. They (strongly) agreed that the PHR was faster (47% inpatients and 45% outpatients) and easier (49% inpatients and 46% outpatients) to use compared to medication reconciliation performed by a healthcare professional (Fig. 3).

## Discussion

In this study, we examined usability and perceived usefulness of an online PHR used for medication reconciliation. Our results show that the usability and perceived usefulness of the PHR was good. There were no patient-, clinical-, hospital-, and ICT-related factors significantly associated with usability.

This study is the first multicenter study assessing usability of a PHR used for detecting medication discrepancies. In two other monocenter studies performed in European hospitals the usability of a PHR for the identification of medication discrepancies has also been studied with the validated SUS [17, 18]. Like in our research, the mean SUS-score (of 68) observed by Webering Buning et al. was classified to the adjective rating ‘good’. Meanwhile, the median SUS-score (of 75) observed by Marien et al. was classified to the adjective rating ‘excellent’ [17, 18]. Compared to our research, Marien et al. used another PHR, included patients who used at least five drugs and the majority of the patients (63%) had a high education level (university of applied sciences or university). Our more diverse study sample may have resulted in a higher external validity and potentially resulted in the inclusion of patients who were less able to use a PHR causing a lower overall SUS-score.

In our research, usability and perceived usefulness were both classified according to the adjective rating scale of Bangor et al. [25]. We observed that most patients classified the usability and perceived usefulness as good. If we look closer to the classification categories, we realized that a score of at least 51 out of 100 points was categorized as good. However, it should be questioned if this actually reflects a good usability and perceived usefulness as just half of the points were scored. As we observed that the majority (83%) of the patients classified the perceived usefulness as poor, ok or good, we assume that perceived usefulness of PHRs used for medication reconciliation should be classified as fair to good instead of good. In our population, only two inpatients and none of the outpatients classified the usability of the PHR as ‘worst imaginable’ or ‘awful’. This skewed distribution of scores towards good usability is potentially related with the fact that patients become more experienced in using digital devices [30, 31]. Besides that, the PHR used in our research is especially designed for patients, and the number of data entries by the patient is limited, increasing the usability of the system.

According to the technology acceptance model, patients’ perceived usefulness and perceived ease of use are the most important factors for adoption of technology devices [16]. If we focus on the items of the perceived usefulness questionnaire which were most relevant for medication reconciliation (including necessary for the visit, increased preparation for the visit and increased time to discuss during the visit), our agreement range (defined as the minimum and maximum proportion of patients who (strongly) agreed with an item of the questionnaire) was higher (39–63%) compared to the agreement range (17–63%) when all items of the perceived usefulness questionnaire were taken into account. This suggested that for medication reconciliation purposes the perceived usefulness of the PHR was acceptable.

In the study of Marien et al. the perceived usefulness of a PHR was higher compared to our results (agreement range of 80–100% versus 17–63%). In the study of Marien, prior to the use of the PHR, patients were educated how to use the PHR and also encouraged to use the system, which was not done in our study [18]. Information provision and support by a healthcare professional are related with a higher intention to use a PHR [32, 33]. So, in order to increase the ease of use and the perceived usefulness by patients, it is recommended that hospitals inform patients about how to use a PHR and about the usefulness of using a PHR.

The difference in perceived usefulness between Marien et al. and our study may also be related with the type of questions: Marien et al. primarily focused on time and communication benefits of the created medication list and we focused on the perceived usefulness in relation to patient’s drug use and the benefits of the current drug list during the hospital visit. Besides that, PHR users who had a negative opinion about the PHR and/or had a suggestion to improve the PHR were potentially more motivated to participate in this study which may have caused inclusion bias. Furthermore, Marien et al. included patients with pulmonary transplants who have potentially a higher perceived usefulness of their (live saving) drugs compared to our study sample [34].

The strength of our study is that we compared usability and perceived usefulness between patient-, clinical-, hospital-, and ICT-related factors at both the in- and outpatient setting. This resulted in a better knowledge about which patients have difficulties in using the PHR and which patients have negative thoughts about the usefulness of the PHR. Until now, only one study investigated the association between patient characteristics and usability. Marien et al. observed an association between the number of logins to the PHR (> 4 times during the study) and usability [18]. As our patient sample had less logins to the PHR, we did not observe this effect. Besides that, we observed that only 9% of the outpatients had a follow-up appointment. Because of this, it is unknown how repeated use influences the usability and perceived usefulness of the PHR. Meanwhile, in the univariate analyse of the in- and outpatient setting, we observed that experience with digital devices was significantly associated with a good usability. Irizarry et al. also concluded that computer skills are an important factor that contribute to successful patient engagement via a PHR [15]. To increase the acceptance and use of the PHR in practice, hospitals should offer education about how to use an online PHR for patients with less experience with digital devices.

We observed a high number of drug changes in the last 12 months in our population, which argues for the necessity of a continuous use of PHRs by patients. However, the number of patients that used the PHR more than once in the last 12 months was small, although the majority of the patients indicated that PHRs are easy to use and useful. In other words, the full benefits of a PHR are not reached and therefore patients should be stimulated to continuously update their drug list in the PHR. This can for example be reached by educating patients about usability and perceived usefulness of PHRs and/or by reminding patients to update their drug list in the PHR. As electronic reminders are positively associated with patient adherence to medication [35], we assume that patients will become more empowered when they are (continuously) reminded to update their own drug list, especially when the number of drug changes is high. Therefore, we recommend that future studies should focus on usability of the combination of electronic reminders with PHRs.

Unlike the usability, there is no standardized method to score perceived usefulness. Because of this, we scored the perceived usefulness in a similar way as the SUS. As there was also no standardized dichotomization for analysing the association between perceived usefulness and patient-, clinical-, hospital-, and ICT-related factors, we compared the first and fourth quartile of the perceived usefulness score. To confirm the robustness of our results, we also performed additional analyses. These analyses did not show any consistent associations between the variables and the perceived usefulness. Because of this, we assume that there is no significant association between the perceived usefulness and patient-, clinical-, hospital-, and ICT-related factors at both the in- and outpatient setting.

Finally, we observed that around half of the patients (strongly) agreed that the PHR was faster and easier to use compared to medication reconciliation performed by a healthcare professional. Therefore, further research should examine the time-advantages and the accuracy benefits of using PHRs for medication reconciliation in usual care.

Our study had several limitations. Firstly, the Dutch version of the SUS questionnaire used in our study has not been validated. Nevertheless, the Dutch version has been widely used and have similar internal reliability to the original English version [18, 24, 36–40].

Secondly, there is a chance of non-response bias. More than two-third of the patients refused to participate in this study which may have affected usability and perceived usefulness of the PHR.

Thirdly, there is a chance of recall bias, because the time between using the PHR and performing the questionnaire was about a month. As Boyd et al. demonstrated that there is no significant change of the user’s recollection of usability of digital product over a period of 6 months [41], we assume that the risk of recall bias is minimal.

Fourthly, there may have been errors in documenting the types of comorbidities based on prescribed medications. However, this should not have led to major errors in the count of comorbidities, which was the covariate used in analyses.

Fifthly, the results of our study with regard to the inpatients may not be generalizable to other countries where the numbers of planned hospitalizations for medication conditions are small.

Sixthly, the questionnaire was only sent to patients who completed the medication check in the PHR. Patients who did or could not use the PHR or patients who quitted halfway through the process due to difficulties were not participating which potentially resulted in an overestimated usability and perceived usefulness of the PHR. As the acceptance rate of the PHR is low (about 44%) [14], the adoption of the PHR should be improved by increasing the information provision (about the usefulness of the PHR and how to use it) to patients and involve patients by improving the design of the PHR. Further research should focus on the barriers and facilitators of a PHR among PHR users and non-users. Knowledge of these barriers and facilitators may contribute to a better targeted approach to improve the adoption of the PHR. Improvement of the PHR may have the potential to positively affect the quality of medication discrepancies detection with the PHR, thereby increasing the success of the PHR.

## Conclusions

In conclusion, the majority of the patients indicated that the PHR used for medication reconciliation was useful and easy to use, but there is still room for improvement to increase the adoption of PHRs for medication reconciliation. To further increase acceptance and use of a PHR, further research should explore the barriers and facilitators of the population who rated the usability and perceived usefulness low.

## Supplementary Information

**Additional file 1.**

**Additional file 2.**

**Additional file 3.**

## Data Availability

The datasets generated and/or analyzed during the current study are not publicly available due to privacy restrictions as the databases contain information that could compromise the privacy of research participants. However, the datasets are available from the corresponding author on reasonable request.

## Abbreviations

IQR: Interquartile range
PHR: Personal health record
SD: Standard deviation
SUS: System Usability Scale
Q1: First quartile
Q4: Fourth quartile
