# Supplementary material for: Usability and perceived usefulness of patient-centered medication reconciliation using a personalized health record: a multicenter cross-sectional study
Source: BMC Health Serv Res. 2022 Jun 13;22:776. doi: 10.1186/s12913-022-07967-7 (PMC9195254; doi:10.1186/s12913-022-07967-7)

Additional file 1: Screen shot of patient views of the personal health record

During the verification process, the personal health record presents the combined information from both the professionals and the patient file in a simple and understandable drug list. Patients are asked to modify or confirm the shown medication information. If there is a difference between the listed drugs and how patients actually use the drugs, patients note the correct dose, formulation, frequency, route of administration and/or strength in their personal health record. Patients are also able to stop medication that is no longer in use and add new medication. Besides that, patients are able to report adverse events and comments related to their reported drug list.

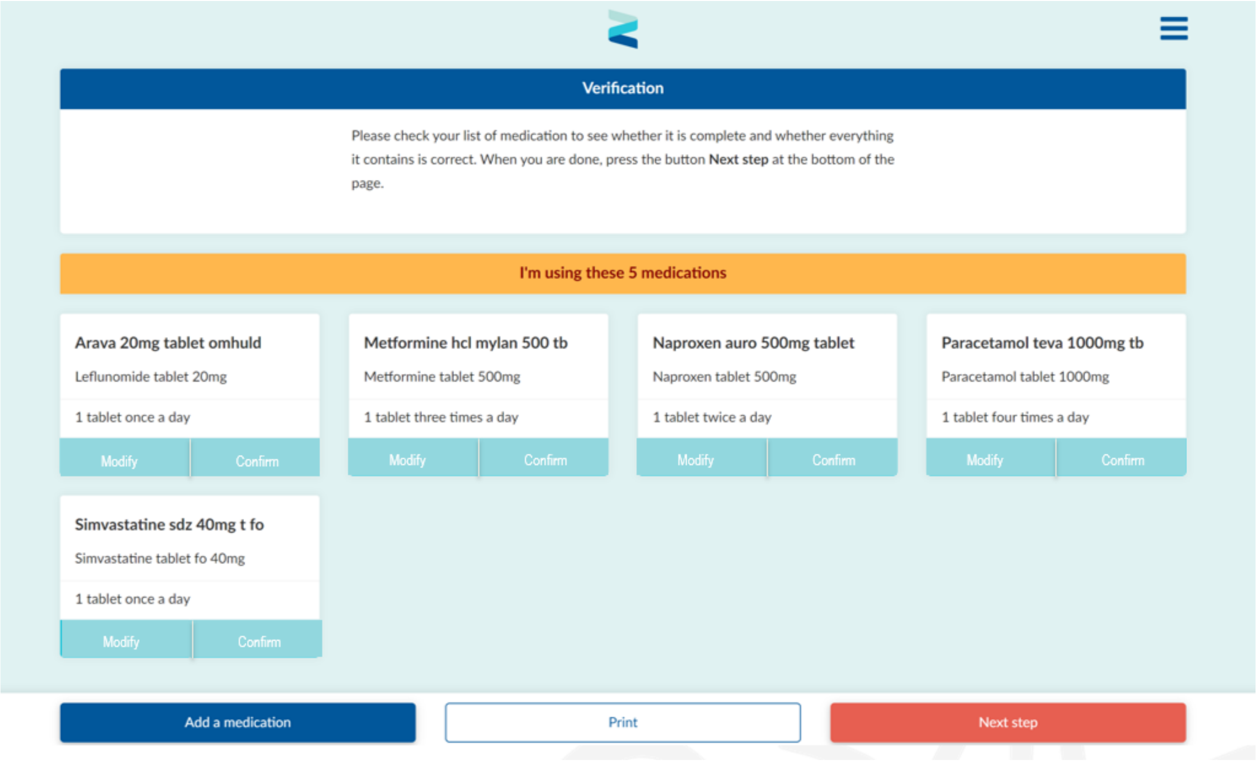

Supplement: Supplementary file 1 — Additional file 1. [file 12913_2022_7967_MOESM1_ESM.pdf]
