# Supplementary material for: Usability and perceived usefulness of patient-centered medication reconciliation using a personalized health record: a multicenter cross-sectional study
Source: BMC Health Serv Res. 2022 Jun 13;22:776. doi: 10.1186/s12913-022-07967-7 (PMC9195254; doi:10.1186/s12913-022-07967-7)
Supplement: Supplementary file 2 — Additional file 2. [file 12913_2022_7967_MOESM2_ESM.pdf]

Additional file 2: Screen shot of the healthcare professional view of the personal health record

After the patient has reported their drug list in the personal health record, the healthcare professional sees the entered information. Per medication, the healthcare professional sees which information is derived from which source (the patient, the hospital system, the Nationwide Medication Record System, and/or the previous validation). If there is a difference between the information reported in these sources, a red bar with ‘make a choice’ is highlited. Subsequently, the healthcare professional determine which information/source indicate the correct drug information. In case of uncertainty, the professional contacts the patient. After touching the correct drug information, an overview of the generated drug list is shown and the healthcare professional can report some comments. Finally, the healthcare professional saves the drug list and the information is processed in the electrectronic health record (in case the personal health record is connected to the electronic health record).

Patient: **Mr. Sven XXX_Vink** Email: **yes** Home: **none** Cell: **0618319030**
Birthdate: **08/18/1954 (67 years)** BSN: **...905** ✓ Ph'cies: **none** Resident of: **Stitswerd**

Overview | **Medicines** | His profile | Access by... | Messages | Print and PDF | SOS-service

Adalimumab injvlst pen 40mg=0,4ml (100mg/ml) — Humira 40 injvlst 100mg/ml pen 0,4ml

**Patient**	Current	1 injection once per week	**01/31/2022**	GB
**Your system**	In the past	—	02/05/2021	GB
**Your validation**	Make a choice			

Asciminib 40mg tabl(gratis)

**Patient**	Current	1 tablet 2 times per day	**01/31/2022**	GB
**Previous validation**	Current	1 tablet 2 times per day	12/20/2021	GB
**Your system**	Current	1 tablet once per day	03/03/2021	GB
**Your validation**	Current	2d1t<br>**Usage:** 1 tablet 2 times per day		

Broomhexine drank 0,8mg/ml — Bisolvon drank voor kinderen 0,8mg/ml aardbeismaak

**Patient**	In the past	—	**01/31/2022**	GB
**Previous validation**	Current	once per day	12/20/2021	GB
**Your validation**	Make a choice			

Darbepoetine alfa injv wwsp 150µg=0,3ml (500µg/ml) — Aranesp 150 injvlst 500µg/ml wwsp 0,3ml

**Patient**	Current	once per week	**01/31/2022**	GB
**Your validation**	Make a choice			

Dextran 70/hypromellose oogdr 1/3mg/ml fl 15ml — Duratears oogdruppels flacon 15ml

**Patient**	Current	Twice a day in the left eye	**01/31/2022**	GB
**Previous validation**	Current	Twice a day in the left eye	12/20/2021	GB
**Your system**	Current	Twice a day in the left eye	03/03/2021	GB
**Your validation**	Current	;Twice a day in the left eye		

Estradiol 400 mcg cap

**Patient**	Current	1 capsule 2 times per day	**01/31/2022**	GB
**Previous validation**	Current	1 capsule once per day	12/20/2021	GB
**Your system**	Current	1 capsule once per day	03/03/2021	GB
**Your validation**	Current	;1 capsule 2 times per day		

Ibuprofen tablet 400mg — Ibuprofen teva tablet 400mg ot

**Patient**	Current	1 tablet 2 times per day	**01/31/2022**	GB
**Previous validation**	Current	1 tablet 2 times per day	12/20/2021	GB
**Your system**	Current	1 tablet 2 times per day	03/18/2021	GB
**Your validation**	Current	2d1t<br>**Usage:** 1 tablet 2 times per day		

Metformine tablet 500mg — Metformine hcl mylan tablet 500mg

**Patient**	Current	1 tablet once per day take with water	**01/31/2022**	GB
**Previous validation**	Current	1 tablet once per day take with water	12/20/2021	GB
**Your system**	Current	1 tablet once per day take with water	03/03/2021	GB
**Your validation**	Current	1d1t mwi<br>**Usage:** 1 tablet once per day take with water		

Methotrexaat tablet 2,5mg — Methotrexaat sandoz tablet 2,5mg

**Patient**	Current	4 tablets once per week take with water on Monday	**01/31/2022**	GB
**Previous validation**	Current	4 tablets once per week take with water on Monday	12/20/2021	GB
**Your system**	Current	4 tablets once per week take with water on Monday	12/13/2019	GB
**Your validation**	Current	mtx4 ma<br>**Usage:** 4 tablets once per week take with water on Monday		

Metoprolol tablet mga 100mg (succinaat) — Metoprololsuccinaat mylan ret 100 tabl mva 95mg

**Patient**	Current	1 tablet 2 times per day take with water	**01/31/2022**	GB
**Previous validation**	Current	1 tablet 2 times per day take with water	12/20/2021	GB
**Your system**	Current	1 tablet 2 times per day take with water	02/05/2021	GB
**Your validation**	Current	2d1t mwi<br>**Usage:** 1 tablet 2 times per day take with water		

Paracetamol tablet 1000mg — Paracetamol teva tablet 1000mg

**Patient**	Current	when necessary 1 tablet twice a day	**01/31/2022**	GB
**Previous validation**	Current	when necessary 1 tablet twice a day	12/20/2021	GB
**Your system**	Current	when necessary 1 tablet twice a day	10/30/2020	GB
**Your validation**	Current	ZN ;1 tablet twice a day<br>**Usage:** when necessary 1 tablet twice a day		
