# Supplementary material for: Usability and perceived usefulness of patient-centered medication reconciliation using a personalized health record: a multicenter cross-sectional study
Source: BMC Health Serv Res. 2022 Jun 13;22:776. doi: 10.1186/s12913-022-07967-7 (PMC9195254; doi:10.1186/s12913-022-07967-7)
Supplement: Supplementary file 3 — Additional file 3. [file 12913_2022_7967_MOESM3_ESM.pdf]

Additional file 3: Characteristics of PHR users and non-users at the inpatient and outpatient setting

In totally, 1221 inpatients and 5282 outpatients were invited to perform MR by using a PHR. An independent-samples nonparametric test and Chi-square test were used to compare the age and gender of the PHR users and non-users.

	Inpatients (n = 1221)		Outpatients (n = 5282)	
	**PHR-users (n = 522)**	**PHR non-users (n = 699)**	**PHR-users (n = 2425)**	**PHR non-users (n = 2857)**
**Male (%)**	63.0	63.9	36.6	36.0
**Age in years, median (IQR)**	65 (56-72)*	68.0 (57-74)	58.0 (48-67)*	55.0 (43-67)

*$P < 0.05$
